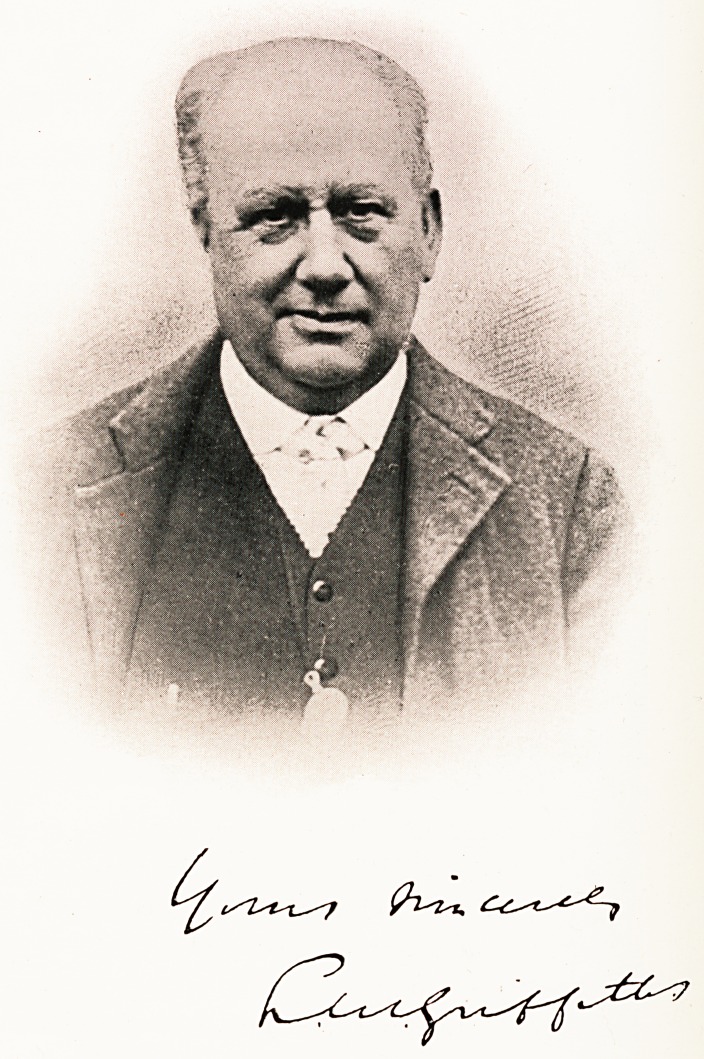# Lemuel Matthews Griffiths

**Published:** 1925-01

**Authors:** 


### Obituary.

### LEMUEL MATTHEWS GRIFFITHS, M.R.C.S., L.R.C.P.

When we think of Lemuel Matthews Griffiths, whose death on September 22nd, 1924, at the ripe age of 79 we all deplore, we may well recall the remark which Samuel Johnson once made with regard to another eminent character closely associated with our city: "If a man were to go by chance at the same time as Burke under a shed, to shun a shower, he would say, 'This is an extraordinary man.'" But it was only those who were brought into close contact with him who could fathom the workings of his mind and recognise the latent powers of an unique personality, a feeling admirably expressed by the motto from one of the sonnets of his great earthly master, Shakspere, which accompanied a wreath of bay leaves placed on his coffin :

> " Who is it that says most ? Which can say more,
> Than this rich praise, that you alone are you ? "

It would take more than a mere obituary notice to dwell on the many-sidedness of the man and on his abounding energy, his perseverance and the immense amount of work which he quietly accomplished, many of the fruits of which we as a Society have fully reaped and which have influenced the lives of many others. Not only a busy medical practitioner of the old school, whose skill was highly appreciated, he was a trusty family friend whose advice was always valued and in whom confidence was never misplaced. It has been well said that, after all, the most living of the gifts of Greek medicine to our time is its ethical element, and no one acted up to the spirit of the oath of the great father of medicine better than L. M. Griffiths.

To appreciate his other numerous activities it is necessary to give a brief sketch of his life. Born in 1845, he lost both his parents in early childhood. His school days were passed in Somerset Street, Kingsdown, under Mr. Vines, a well-known schoolmaster of the time, and he received his medical training at the Bristol Medical School. He took his M.R.C.S. Eng. in 1867 and L.R.C.P. Edin. in 1868, and at once went into general practice in the Hotwells. He held various medical appointments, viz. Medical Examiner and School Medical Officer of Mentally Defective Children under the Bristol Education Committee, Consulting Surgeon to the Clifton Dispensary and Medical Officer to Lady Haberfield's Alms Houses.

From 1890–93 he was Hon. Librarian to the Library of the Bristol Medico-Chirurgical Society and from 1893–1902 to the Bristol Medical Library, and for many years Assistant Editor of the *Bristol Medico-Chirurgical Journal*. In 1887 he was elected President of the Society. He was a born librarian, and anything in the shape of a book appealed to him, and his connection with the Medical Library was the happy means of conferring the greatest boon on the practitioners of Bristol and its neighbourhood ; for it was mainly due to his efforts that the splendid collection of books, which has lately been handed over to the University, was got together. Whenever any one of our profession, who remembers the debt owed to him, enters their handsome new quarters " Si monumentum requiris, circumspice " will involuntarily come to his lips. The Medico-Chirurgical Society elected him an Honorary Member, the highest recognition of his great services that it was in their power to confer.

Mr. Griffiths's interest in anything relating to the medical history of Bristol was shown by his valuable contributions to the *Bristol Medico-Chirurgical Journal*, which includes papers on "The Reputation of the Hotwells as a Health Resort," "The Bristol Medical Reading Society" and "The Bristol Medical Library," while in the *Library Association Record* he published a paper "On some things of general interest in the Bristol Medical Library." He also supplied from time to time valuable notes on Medical Philology to the *Journal*. These he published separately in a small volume in 1905.

In 1887 he contributed an interesting article on "Shakspere and the Medical Sciences." This leads one naturally to refer to his life-long study of Shakspere and his times. In 1889 he published his *Evenings with Shakspere*, a most useful and carefully prepared volume, which has proved of invaluable service to Shakspere Societies in various parts of the world, and on which the Clifton Shakspere Society, of which he was the founder and first Secretary and Librarian, was modelled. By his indefatigable industry and perseverance, and by getting into touch with many eminent Shaksperians and securing their interest, he placed it on a firm basis and collected for it a most useful library, and it is now flourishing in the year of its jubilee.

His private library was one of the largest in Bristol, and included works on the most miscellaneous subjects, being especially rich in maps and books relating to the city; but, after all, his chief love was for Shakspere and Elizabethan literature generally, the term being used in the widest sense—every room in his house, including his bathroom, was full of books, and even the tables, sofas and chairs were piled with them, but the most sacred room of all was one entirely devoted to the great poet and his age. Mottoes from his works adorned the vacant spaces on the walls, and it was here that he loved to spend his evenings and entertain his friends.

It has been well said that one of the subsidiary sorrows of a scholar's death is the dispersal of his books, but in this case the regret is mitigated by the thought that so many of Mr. Griffiths's private friends and public institutions, including the Bristol University, Central Library and Art Gallery, as well as the British Museum and Birmingham Shakspere Library, have benefited by his thoughtful care. One of his chief hobbies was the collection of magazine articles on various subjects and having them bound and indexed in many volumes, and it was one of his greatest pleasures to send from time to time to many of his friends rolls of cuttings, carefully selecting only those which he thought would especially interest them.

For many years, up to shortly before the time of his death, Mr. Griffiths spent his leisure moments on what he regarded as his "opus magnum," which was no less than the illustration of *The Dictionary of National Biography* by portraits of as many of the characters included in it as he could find. He managed to get together thousands of them from all sorts of sources, which he carefully placed in a huge folio which he has bequeathed to the National Portrait Gallery.

Amid all his labours of a professional and literary character he found time to devote himself to much social and religious work. A devoted son of the Church of England, and conscious of ever being in his great Task-Master's eye, he spent an immense amount of time in what he probably regarded as intrinsically of far more value. For many years he took a keen and practical interest in Holy Trinity Church, Hotwells, founding the Sunday School for Men and becoming the intimate friend of the late Canon Wallace. He paid frequent and regular visits to the Clergy Daughters' School and the Gordon Home for Blind Women and read and talked to the inmates. He was closely associated with the Cathedral Branch of the Church of England Men's Society, and among the poor and outcast of St. Jude's he laboured incessantly. No figure was more familiar than his in the common lodging houses of the city, and he had a wonderful power of getting hold of the men and influencing them for good. In order to place himself more in touch with them he instituted an entertainment on one evening a month during the winter. Nor did he forget the women. A devoted band of ladies undertook to meet them once a week to help them in various ways. Every Sunday evening for many years he held a service for men in Wade Street, and gathered round him many of these derelicts and took an interest in their lives. But perhaps his most useful work was in establishing the Men's Night School. Hundreds of men, many of whom could neither read nor write, attended at one time or another the evening classes, which met weekly during the winter months; and in the summer he invited them to tea and amusements in his garden. He had a wonderful way of attracting helpers around him, encouraging them with his high ideals and making them all feel happy and enthusiastic in their work. His memory is still cherished by many a poor working man, and the feeling which he inspired among all his friends cannot be better expressed than by the lines of the great dramatist:—

> "The dearest friend to me, the kindest man,
> The best conditioned and unwearied spirit
> In doing courtesies."

## Figures and Tables

**Figure f1:**